# Tissue Is More Important than Time in Stroke Patients Being Assessed for Thrombolysis

**DOI:** 10.3389/fneur.2018.00041

**Published:** 2018-02-05

**Authors:** Andrew Bivard, Neil Spratt, Ferdinand Miteff, Christopher Levi, Mark William Parsons

**Affiliations:** ^1^Department of Neurology, John Hunter Hospital, University of Newcastle, Newcastle, NSW, Australia

**Keywords:** acute stroke, brain imaging, thrombolysis, brain ischemia, cerebral blood flow, cerebrovascular disease

## Abstract

**Aim:**

The relative prognostic importance of modern imaging profiles compared with standard clinical characteristics is uncertain in acute stroke patients. In this study, we aimed to compare baseline multimodal CT imaging measures with known clinical predictors of patient outcome at 3 months [modified Rankin scale (mRS)].

**Methods:**

We collected baseline, 24 h, and day 90 clinical and imaging data from acute ischemic stroke patients being assessed for thrombolytic therapy between 2010 and 2015 at a single center as part of a retrospective analysis.

**Results:**

561 patients presenting within 4.5 h of ischemic stroke onset who were eligible for thrombolysis based on standard clinical criteria were assessed. Acute infarct core volume on CTP was the strongest univariate predictor of patient outcome (mRS 0–2, *R*^2^ 0.497, *p* < 0.001), followed by collateral grade (mRS 0–2, *R*^2^ 0.281, *p* < 0.001). The strongest baseline clinical predictor of outcome was National Institutes of Health Stroke Scale (NIHSS) (mRS 0–2, *R*^2^ = 0.203, *p* < 0.001). Time to treatment (mRS 0–2, *R*^2^ 0.096, *p* = 0.01) and age (mRS 0–2, *R*^2^ 0.027, *p* = 0.013) were relatively weak univariate baseline clinical predictors of 3-month outcome. In multivariate analysis, acute infarct core volume and collateral grade were the only significant baseline predictors of 3-month disability (both *p* < 0.001).

**Conclusion:**

In patients assessed for thrombolysis by combined clinical and multimodal CT criteria within 4.5 h of onset, the size of the CTP infarct core and collateral grade on multimodal CT were highly predictive of patient outcome. Standard clinical variables, including time to treatment and NIHSS, were not as strongly predictive as multimodal CT variables.

## Introduction

The meta-analysis of clinical trials of intravenous thrombolytic therapy for ischemic stroke demonstrates declining odds of favorable outcome with time since symptom onset (1). The magnitude of this effect, however, is relatively small, and although rapid workflows should not be discouraged, other influences on outcome such as infarct core volume require further exploration. Multimodal CT imaging (incorporating CTA and CTP) before stroke thrombolysis can reliably identify the extent of irreversibly damaged tissue and the likelihood of responsiveness to thrombolytic therapy (2–5). It is well recognized that significant heterogeneity exists in cohorts of acute stroke patients, and the different subgroups of patients demonstrate a wide range of responsiveness to intravenous thrombolysis (6, 7). Indeed, some subgroups of patients show very limited or no benefit from thrombolysis (8). Despite this information, the relative prognostic influence of pretreatment advanced imaging variables (e.g., infarct core, collateral status, and penumbral volumes) compared with standard clinical predictors [e.g., age, NIHSS, and time from symptom onset (1)] is uncertain. In some situations, this can lead to an overemphasis or even a sole emphasis on time from symptom onset when determining suitability for intravenous thrombolysis.

Our primary aim was to identify if multimodal CT measures was a significantly stronger predictor of patient outcome than standard clinical variables such as time to treatment and baseline stroke severity in patients treatment with intravenous thrombolysis. We hypothesized that multimodal CT imaging predictors of clinical outcome would be an order of magnitude greater in predicting outcome compared with baseline clinical measures (including time to thrombolytic treatment).

## Patients and Methods

### Patients

We prospectively collected baseline, 24 h, and day 90 clinical and imaging data from all acute ischemic stroke patients being considered for acute thrombolytic therapy (2010–2015) for analysis retrospectively. All potential thrombolysis patients presenting within 4.5 h of symptom onset were screened on arrival by a stroke neurologist, and if they had an acute neurological deficit deemed significant enough to warrant consideration for thrombolysis and were not likely to be a mimic or TIA, they underwent non-contrast CT (NCCT), CTP, CTA, and 24-h follow-up imaging with magnetic resonance imaging (MRI). For this study, lacunar and basilar strokes as well as patients with a premorbid mRS of 2 were excluded. Clinical stroke severity was assessed immediately prior to acute and 24-h imaging using the National Institutes of Health Stroke Scale (NIHSS). At 90 days after stroke, patient disability was assessed by the modified Rankin scale (mRS) by certified research coordinators who were blind to baseline clinical and imaging data (in cases where clinic attendance was not possible, mRS was determined by standardized telephone assessment) (9). Endovascular clot retrieval was not available at the study center during the recruitment period. All data collection was approved by the Hunter New England Heath District ethics committee governed by the Helsinki Declaration of 1975 (and as revised in 1983) and all patients gave written informed consent for the use of their data for research.

#### Multimodal CT “Decision-Assistance” for Thrombolysis

CTP was routinely used as part of the decision-making process in addition to standard clinical criteria for thrombolysis (10). The CTP criteria for treatment were based on qualitative assessment of the vendor software perfusion maps. If patients fulfilled standard clinical and NCCT criteria for treatment but demonstrated any of the imaging characteristics listed below, they were considered less ideal candidates for thrombolysis and, in individual cases, the treating clinician may have chosen to withhold rtPA treatment:

1. Absent or very small perfusion lesion (on transit time maps) qualitatively assessed at less than 15 mL.
2. An infarct core on CTP (determined by qualitatively low CBV and CBF) larger than 1/3 middle cerebral artery (MCA) territory (or >1/2 anterior or posterior cerebral artery territory), even if NCCT did not show the same extent of early ischemic change.
3. Lack of definite visual “mismatch” between the transit time lesion and the CBV and CBF lesions, indicating lack of potentially salvageable tissue.

We did not use specific CTA criteria for guidance on rtPA treatment eligibility in anterior circulation stroke syndromes.

### Multimodal CT Protocol

Acute imaging included whole brain NCCT, CTP, and CTA using a 64-slice scanner (64-slice Philips Brilliance). NCCT was followed by perfusion CT, comprising two 60-s series with 40-mL contrast agent (Ultravist 370; Bayer HealthCare, Berlin, Germany) injected at 6 mL s^–1^ followed by 30 mL of saline at 6 mL s^–1^. CT angiography was performed after perfusion CT with acquisition from the aortic arch to the top of the lateral ventricles (11) with a second contrast injection of 40-mL contrast (Ultravist 370; Bayer HealthCare, Berlin, Germany) injected at 6 mL s^–1^ followed by 30 mL of saline at 6 mL s^–1^.

### 24-h Imaging Protocol

All patients, regardless of treatment, underwent an MRI scan with a stroke protocol on a 1.5T or 3T scanner (Siemens Avanto or Verio). The MR protocol included the following: diffusion-weighted imagine (DWI), perfusion-weighted imaging (PWI), and MR time of flight angiography (TOF). For those with a contraindication to MRI, repeat NCCT and CTP were performed using the above protocols.

### Image Post-Processing

For the outcome analyzes, imaging post-processing was with commercial software for quantitative analysis (MiStar, Melbourne, Australia). Perfusion data were processed by a single-value deconvolution algorithm with delay and dispersion correction (12). Previously validated thresholds were applied in order to measure the volume of the acute perfusion lesion (relative delay time 3 s) and acute infarct core (relative CBF < 30%) (13).

Leptomeningeal collateral ratings and symptomatic large vessel occlusion status on baseline CTA were performed by two stroke neurologists, with any disagreement resolved by consensus with a third neurologist. The determination of whether contrast in the territory distal to the occlusion was filling antegrade or retrograde was conducted using dynamic (multiple time point) CTP source images to determine if there was partial occlusion (antegrade flow observed) or complete (i.e., only retrograde flow *via* collaterals observed) (14). A 3 point-grading scale was used for occlusion (none, partial, complete). Collateral grade was determined by assessment of the extent of reconstitution of contrast distal to a complete occlusion on CTA using the Mitef score (a 3-point scale: good, moderate, or poor) (15). ASPECTS “infarct core” scores were recorded on NCCT, CTA source images, and on the CTP CBV maps (16, 17). Symptomatic intracranial hemorrhage (sICH) was determined by the presence of parenchymal hematoma type 2 and NIHSS worsening by four or more points at 24 h (18).

### Statistical Analysis

To determine predictors of 3-month disability, we analyzed patients who had CTP used as part of the thrombolytic treatment decision-making process. All patients presenting within 4.5 h of symptom onset, being considered for IV rtPA with hemispheric ischemia were assessed for this study. Patient characteristics which were continuous variables were compared between treated and untreated groups using the students *T*-test when data were normally distributed or the Wilcoxon rank-sum when the data were not normally distributed and data presented as mean and SD or as median and inter-quartile range (IQR) where appropriate. Categorical variables were compared using chi-square test. We also performed a subgroup analysis consisting of the patients treated with IV rtPA. Clinical and imaging data were used as independent variables to predict 3-month outcome with regression analyses (SPSS version 20, IBM). First, univariate regression was performed to determine the relationship between clinical and imaging variables with 3-month mRS, dichotomized at 0–2 vs. 3–6, in all patients being considered for thrombolysis, and then again in the subgroup of patients treated with IV rtPA. Data were also presented in table format which included the R and coefficients from the linear regression analysis. All patients analyzed were required to have a premorbid mRS of 2 or less.

Imaging variables which were assessed in the univariate analysis were as follows: acute perfusion lesion volume, acute infarct core volume, acute penumbral volume, mismatch ratio, ASPECTS “core” estimates on NCCT, CTA, and CTP source images, CTA clot location, collateral status, and vessel occlusion grade. Clinical variables that were assessed in univariate analysis were as follows: age, diabetes, smoking, atrial fibrillation, hypercholesterolemia, thrombolytic treatment, time from stroke onset to CT, time from stroke onset to thrombolytic treatment, and acute NIHSS score. Additional analyzes were performed to assess potential interactions between imaging variables and various patient outcome on the mRS.

Next, two multivariate regressions were performed, the first using a backward stepwise approach was performed with the variables of time to imaging, acute NIHSS, age, baseline occlusion, ASPECTS core estimates, acute core volume, acute penumbra volume, CTA clot location, and collateral grade. The backward multivariate regression was also rerun with time to treatment as a variable rather than time to imaging. A second multivariate logistic regression model was generated which did not include multimodal variables. All regression models were performed on all patients, and a second on only rtPA-treated patients. Finally, as a secondary analysis we were also interested in examining the relative effects on clinical outcome of baseline clinical factors (onset time to imaging) vs. baseline advanced imaging variables (infarct core volume). This was assessed by stratifying the patient population by time to imaging at hourly cut points (0–1, 1–2, 2–3, 3–4, and 4–4.5), and infarct core volume divided by 10-mL strata up to 60 mL (0–10, 10–20… 50–60, and 60+).

## Results

### Patients

**Figure 1:**
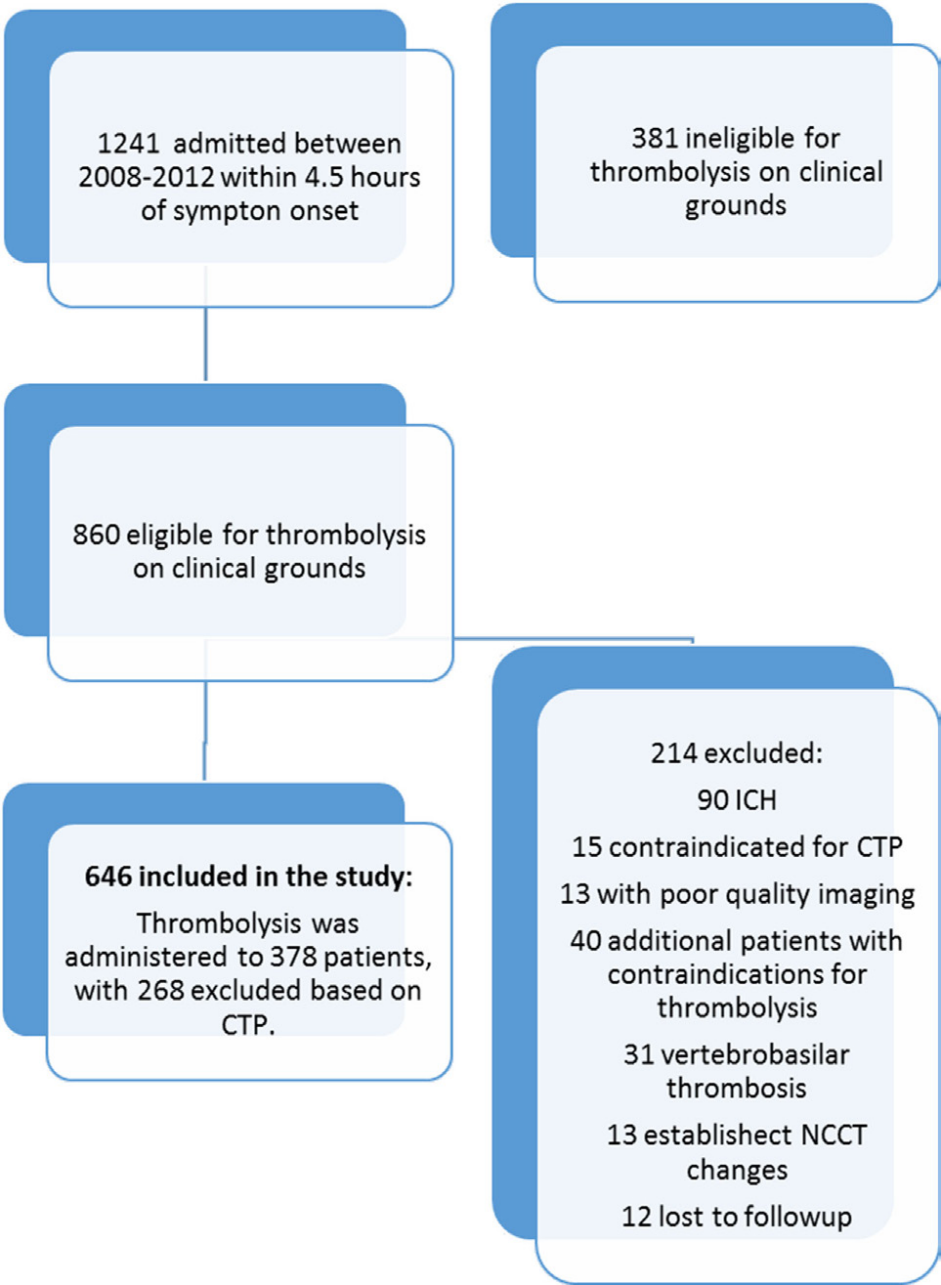
Recruitment flowchart for the study.

Over the study period, 1,241 sub-4.5-h patients presenting with stroke-like symptoms were assessed. On initial neurological triage 381 patients were excluded from this study as being ineligible for thrombolysis based on standard clinical criteria, such as resolving or clinically “minor” deficit, stroke mimic, and significant premorbid disability (mRS > 2). Following patient exclusions, 646 patients with hemispheric ischemic stroke and complete clinical and imaging follow-up who were deemed clinically eligible for rtPA were included in the study. A further 214 were excluded based on thrombolysis eligibility grounds (Figure 1). Of the remaining 646 patients, thrombolysis was administered to 376 patients, and 270 (41%) patients potentially eligible for rtPA based on standard clinical and NCCT grounds did not receive thrombolysis, after visual assessment of perfusion CT was taken into consideration. Baseline characteristics of the treated and untreated groups are shown in Table 1. Of the patients excluded from treatment based on CTP, 31 patients had a visually large ischemic core, 89 patients had a small or no perfusion lesion, and 65 patients were considered to lack significant mismatch visually. The remaining 83 patients were not treated due to standard clinical thrombolysis contra-indications such as a high premorbid mRS or patients being on oral anticoagulation.

**Table 1:** Patient demographics.

	All patients (*N* = 646)	rtPA treated (*n* = 376)	Untreated (*n* = 270)	*p*
Median age (IQR)	73 (51–97)	69 (58–86)	70 (50–97)	*p* = 0.551
Median time to scan (IQR)	171 (106–318)	139 (84–217)	211 (125–327)	*p* = 0.141
Median acute NIHSS (IQR)	12 (5–17)	13 (6–18)	10 (5–14)	*p* = 0.164
Acute core volume (mean, SD)	27.1 (38.5)	28.9 (37.0)	24.7 (40.4)	0.7010
Acute perfusion lesion volume (mean, SD)	81.3 (76.0)	99.8 (70.7)	56.7 (76.0)	<0.001
Acute penumbra volume (mean, SD)	54.1 (52.0)	71.0 (51.0)	31.7 (44.5)	<0.001
Large vessel occlusion (MCA, M1 or ICA, %)	267 (42)	245 (67)	70 (26)	<0.001

*Baseline characteristics for the whole study cohort the characteristics for patients stratified by treatment or not. The p-value represents statistical testing between the values of rtPA-treated and -untreated patient groups*.

### Clinical and Imaging Predictors of Outcome

**Figure 2:**
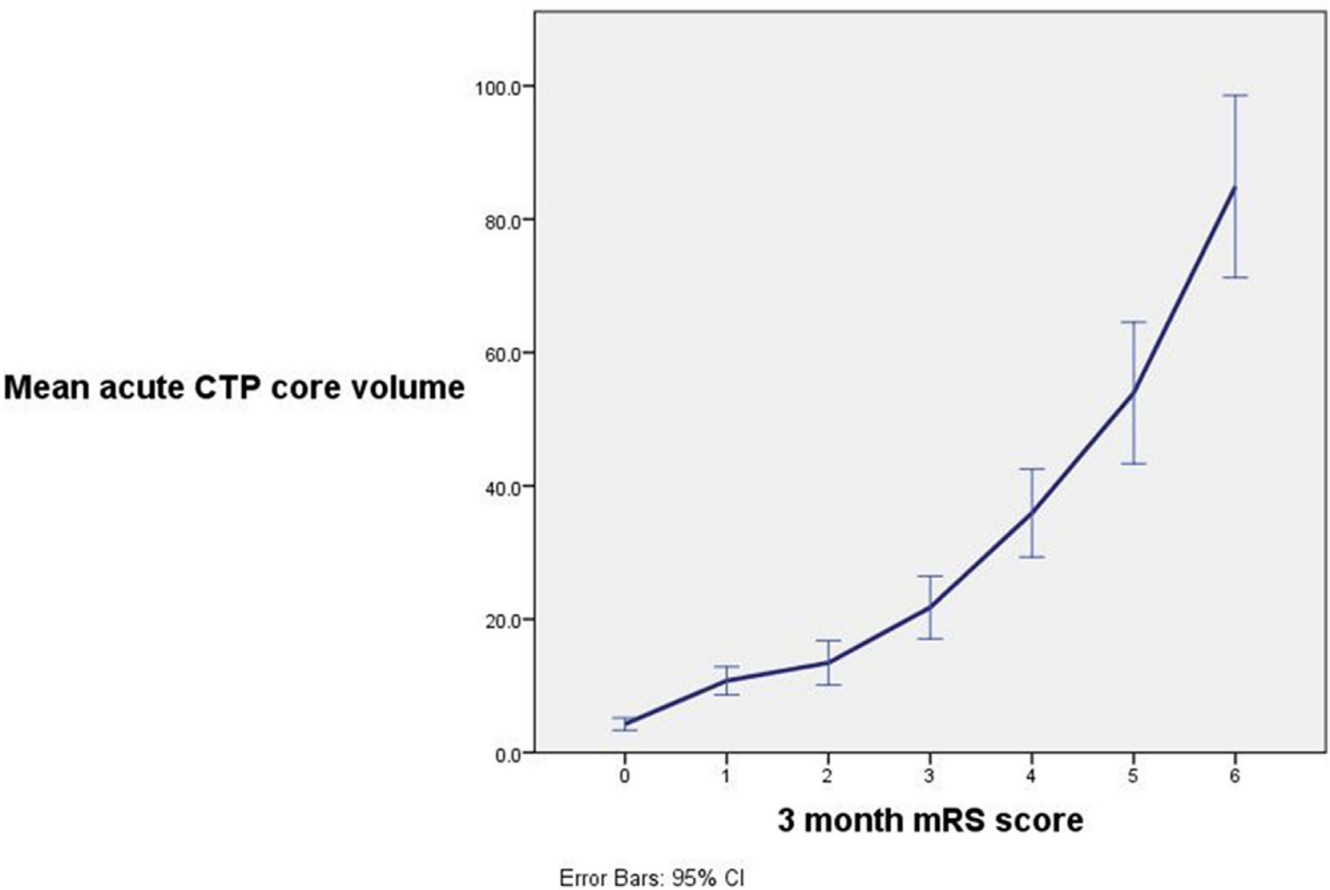
A strong relationship between ischemic core volume and 3-month patient outcome was observed with a decreasing chance of excellent patient outcome with increasing acute ischemic core volume. Note that mean ischemic core was significantly greater between patients with outcome modified Rankin scale (mRS) 0 vs. mRS 1 (*p* < 0.001), mRS 1 and 2 (*p* = 0.006), mRS 3 and 4 (*p* < 0.001), mRS 4 and 5 (*p* < 0.009), and mRS 5 and 6 (*p* < 0.001).

Acute infarct core volume on CTP was the strongest univariate predictor of patient outcome in all patients (mRS 0–2 *R*^2^ 0.497, *p* < 0.001) as well as for those receiving IV rtPA (mRS 0–2 *R*^2^ 0.442, *p* < 0.001, Table 2, Figure 2). The next strongest predictor of 3-month outcome was the baseline collateral grade (all patients mRS 0–2 *R*^2^ 0.281, *p* < 0.001, IV rtPA patients mRS 0–2 *R*^2^ 0.344, *p* < 0.001). CTA clot location was also strongly related to patient outcome (all patients mRS 0–2 *R*^2^ 0.318, *p* < 0.001, IV rtPA patients mRS 0–2 *R*^2^ 0.322, *p* < 0.001). ASPECTS core estimate on any modality was much weaker than CTP core volume in predicting outcome (NCCT ASPECTS: all patients mRS 0–2 *R*^2^ 0.104, *p* < 0.001, IV rtPA patients mRS 0–2 *R*^2^ 0.002, *p* < 0.001). Time to CT (all patients mRS 0–2 *R*^2^ 0.058, *p* = 0.009, Table 3), time to treatment (IV rtPA patients mRS 0–2 *R*^2^ 0.096, *p* = 0.01), NIHSS (IV rtPA patients mRS 0–2 *R*^2^ 0.116, *p* < 0.001), and age (all patients mRS 0–2 *R*^2^ 0.027, *p* = 0.013) were also relatively weak univariate baseline clinical predictors of 3-month outcome.

**Table 2:** Analysis of baseline imaging variables compared with clinical outcome.

	3-month mRS 0–2 in all patients (*n* = 646)	3-month mRS 0–2 in rtPA patients (*n* = 376)

	*R*^2^ (coefficient, *p*)	*R*^2^ (coefficient, *p*)
Perfusion lesion volume	0.198 (0.192, *p* < 0.001)	0.092 (0.208, *p* < 0.001)
Penumbral volume	0.113 (0.144, *p* < 0.001)	0.016 (0.187, *p* < 0.001)
Baseline core	0.497 (0.512, *p* < 0.001)	0.442 (0.456, *p* < 0.001)
Mismatch ratio	0.047 (0.211, *p* = 0.051)	0.071 (0.127, *p* < 0.001)
NCCT ASPECTS	0.104 (−0.218, *p* < 0.001)	0.002 (−0.272, *p* < 0.001)
CTA clot location	0.318 (0.227, *p* < 0.001)	0.322 (0.286, *p* < 0.001)
CTA SI ASPECTS score	0.059 (−0.283, *p* = 0.031)	0.014 (−0.318, *p* = 0.022)
CTP ASPECTS core score	0.243 (−0.181, *p* < 0.001)	0.013 (−0.271, *p* < 0.001)
Baseline occlusion severity	0.155 (0.411, *p* < 0.001)	0.081 (0.328, *p* < 0.001)
Baseline collateral grade	0.281 (0.342, *p* < 0.001)	0.344 (−0.421, *p* < 0.001)

*Univariate analysis of patient 3-month patient outcomes with advanced imaging measures. Penumbral volume was calculated from the volume of the perfusion lesion minus the volume of the infarct core. Mismatch ratio was calculated from the ratio of the perfusion lesion to the infarct core*.

**Table 3:** Analysis of baseline clinical variables compared with clinical outcome.

	3-month mRS 0–2 in all patients (*n* = 646)	3-month mRS 0–2 in rtPA patients (*n* = 376)

	*R*^2^ (coefficient, *p*)	*R*^2^ (coefficient, *p*)
Time to CT (h)	0.058 (0.081, *p* = 0.009)	0.099 (0.131, *p* = 0.002)
Time to treatment		0.096 (0.137, *p* = 0.01)
Acute NIHSS	0.203 (0.119, *p* < 0.001)	0.116 (0.161, *p* < 0.001)
Diabetes	0.001 (0.011, *p* = 0.904)	0.009 (0.027, *p* < 0.001)
Age	0.027 (0.079, *p* = 0.013)	0.019 (0.154, *p* < 0.001)
Hypertension	0.002 (0.021, *p* = 0.238)	0.018 (0.018, *p* = 0.181)
Smoker	0.001 (0.019, *p* = 0.446)	0.001 (0.007, *p* = 0.417)
AF	0.001 (0.091, *p* = 0.451)	0.001 (0.108, *p* = 0.538)
Lipids	0.001 (0.014, *p* = 0.741)	0.001 (0.027, *p* = 0.461)

*Univariate analysis of patient 3-month patient outcomes with clinical assessments*.

Backward multivariate logistic regression was performed to assess the baseline predictors of 3-month outcome using the variables of time to imaging, onset to treatment time, acute NIHSS, age, baseline occlusion grade, ASPECTS core estimates, acute core volume, acute penumbra volume, CTA clot location, and collateral grade. Acute infarct core volume and collateral grade remained significant in the model, which was strongly predictive of mRS 0–2 in all patients (*R*^2^ 0.495, *p* < 0.001) and for those treated with rtPA (*R*^2^ 0.518, *p* < 0.001), while time to imaging, onset to treatment time, acute NIHSS, age, baseline occlusion grade, ASPECTS core estimates, acute penumbra volume, and CTA clot location were not significantly contributing to or dropped out of the model. In another multivariate logistic regression analysis replacing time to CT with time to treatment, time to treatment was not a significant univariate predictor. Interaction testing identified that the acute infarct core volume was significantly associated with 90-day clinical outcome (mRS 0–2 acute infarct core volume covariant −4.1, *p* < 0.0001, Table S2 in Supplementary Material). Additionally, there was no significant interaction between time to imaging and 90-day clinical outcome in the model, which included acute infarct core volume and collateral grade (mRS 0–2, time from symptom onset covariant 0.9, *p* = 0.587, Table S1 in Supplementary Material). Next, in the logistic regression model without multimodal imaging variables, onset to treatment time, acute NIHSS, and age all staid in the model with no variables dropping out; however, the model only had modest predictive of mRS 0–2 in all patients (*R*^2^ 0.244, *p* < 0.001) and for those treated with rtPA (*R*^2^ 0.318, *p* < 0.001).

Time to imaging was weakly correlated with acute core volume (*R*^2^ 0.02, *p* < 0.001), volume of penumbra (*R*^2^ 0.019, *p* < 0.001), and collateral grade (*R*^2^ 0.011, *p* < 0.001). Of note, mean acute core volume was significantly larger for all patients imaged within 3 h (mean acute core volume <3 h, 19.4 mL, *p* < 0.001) compared with those imaged 3–4.5 h after symptom onset (mean 9.6 mL).

## Discussion

The major finding in this study was the strong association between the size of pretreatment infarct core and collateral grade on patient outcome which was an order of magnitude greater than the influence of time to treatment. Importantly, time to thrombolytic treatment was a relatively weak predictor of patient outcome compared with CTP pretreatment infarct core volume and collateral grade. Less-accurate estimates of core (ASPECTS) were also weak predictors of outcome. Multimodal CT infarct core volume and collateral grade were significant multivariate baseline predictors of 3-month clinical outcome in our dataset with time to scan (or time to treatment), age, acute NIHSS, baseline occlusion status, acute penumbra volume, and ASPECTS dropping out of the model despite being significant predictors of patient outcome in univariate analysis. Although the effect of earlier time to thrombolytic treatment on outcome is indisputable from the pooled RCT analyses (19), our data suggest that infarct core volume and collateral grade are likely to be several orders of magnitude greater in influencing the probability of a good outcome following rtPA treatment. These results are in line with those of endovascular patients where time to revascularization has been shown to be of less importance in comparison to other advanced imaging measures for patient treatment suitability (20).

Stratification of our data by baseline CTP infarct core volume identifies that patients with a large ischemic core may not have good outcomes after intravenous thrombolysis. While this has previously been reported, this information is important in the context of time to treatment, as many centers are avoiding performing CTP in favor of (marginally) quicker treatment times. The volume of the acute infarct core did not increase within the 0–4.5-h window by time strata, and yet was a highly significant predictor of outcome, suggesting that time after stroke onset is not a very useful marker of tissue pathophysiology (particularly infarct core). This means that multimodal CT can provide just as much crucial prognostic information within 3 h of stroke onset, as it can beyond. The concept of the infarct core being irreversibly injured tissue likely explains why it is directly related to the 3-month outcome, as with greater tissue loss there is a proportional loss of function, although of course, infarct topography has an important influence (21). The volume of the infarct core has also been identified as a marker of subsequent hemorrhage (22) which further adds to the importance of performing multimodal CT for patient assessment in order to avoid harmful treatment as well as select patients for treatment who will most likely benefit.

The volumes of the ischemic core, collateral status, and the penumbra are intertwined. A small ischemic core (in relation to the total perfusion lesion) requires good quality collateral flow in order to prevent rapid infarct expansion, thus sustaining tissue within the penumbral perfusion threshold, at least until reperfusion can salvage the threatened tissue. Conversely, a large ischemic core would more than likely be the result of poor collateral flow, leading to rapid tissue death and minimal residual penumbra for reperfusion therapy to target. The clinical severity in an individual patient is the result of the entire perfusion deficit, which incorporates the penumbra and core, because both these regions have perfusion below the threshold to support normal neuronal activity. As shown in this study, clinical severity was not an independent multivariate predictor of outcome after thrombolysis. Therefore, the results of this study are supported by sound pathophysiological principles, and the significant individual patient variation in core volume (that cannot be estimated by surrogate measures like time after stroke onset, or NIHSS) means that multimodal CT provides clinicians with more information about the potential for individual patient benefit after intravenous therapy than do clinical variables + NCCT.

This single-center study was performed where patient selection for treatment with IV rtPA is routinely performed using multimodal CT imaging with perfusion and angiography. Patient profiling in this way aims to target patients with a favorable imaging pattern, such as a large volume of penumbra and a small ischemic core, for treatment under the assumption that they have the most to gain from therapy and are the least likely to be harmed (23). The methods used for this study to process CTP data have been validated to identify acute tissue pathophysiology and are being testing in clinical trials (12, 24). However, there is currently no level-I randomized trial evidence supporting decision assistance with multimodal imaging in routine standard practice, despite positive trials of IV thrombolysis (24) and endovascular therapy using this selection approach (25, 26) to target treatment responders. It is important to also note that the effect on this study dataset was that advanced imaging interpretation led to up to 45% of patients being excluded from treatment due to a large ischemic core or lack of salvageable tissue (including those with a small perfusion) (4, 23). This may have lessened the effect of time on outcome as it may have led to more “ideal” patients being treated in the later time windows.

Some important limitations of our study require acknowledgment; this includes an observational, non-randomized design, and its underpinning in pragmatic clinical practice. Next, this study was conducted at a center which uses multimodal imaging in the assessment of patients for thrombolysis therapy which may have altered the patient population to bias the results in favor of imaging variables and produced data which would be different from a center which does not employ sophisticated imaging to select patients for therapy. Variation in the post-processing of imaging may have an impact on previous study results (12). We performed this study using in single, well-validated imaging post-processing platform in an attempt to control for this variation (13). This study used only a single center where advanced imaging is routinely performed for thrombolysis decision assistance. There is the strong possibility in our study of selection bias introduced prospectively with the use of qualitative interpretation of advanced CT imaging during the complex clinical decision-making involving IV tPA administration. This is not possible to control for during retrospective analysis. Next, this study may have been underpowered to show significant relationships with smaller effect sizes (e.g., time to treatment). It is worthwhile noting that a much larger sample size (in meta-analysis) was required than that of the current study to show the effect of time to treatment on outcome (1). Lastly, during this study endovascular therapy was not available; a similar study with such patients may yield different results (27).

This study indicates that within the current <4.5-h time window for rtPA treatment, the volume of the acute core on CTP and collateral grade is a much stronger predictor of outcome than any of the other clinical and imaging variables tested. This applied to both treated and untreated patients. While we agree that patients should be treated as quickly as possible, our results indicate valuable information implying outcome prediction can be obtained by measuring pretreatment tissue and collateral status on multimodal CT. Our data suggest that the time-to-treatment effect is not as a strong a predictor of outcome as are advanced imaging measures. Indeed, time to treatment is probably a poor surrogate for these advanced imaging measures (28). Efforts should also be made to replicate the current findings in a larger multicenter cohort of ischemic stroke patients assessed with multimodal imaging. Patient selection is particularly relevant where assessment for the presence of a large vessel occlusion dichotomizes clinical populations to either thrombectomy or intravenous thrombolysis alone. Therefore, multimodal imaging selection is increasingly being used to assess patients. Therefore, focus should also be placed on streamlining routine multimodal CT assessment in acute stroke patients so as not to delay thrombolytic treatment but to ensure optimal patient selection.

## Ethics Statement

All data collection was approved by the Hunter New England Heath District ethics committee governed by the Helsinki Declaration of 1975 (and as revised in 1983) and all patients gave written informed consent for the use of their data for research.

## Author Contributions

AB, CL, and MP contributed to the concept and design of the study, study funding, acquisition and analysis of data, and drafting the manuscript and figures. NS and FM contributed to acquisition and analysis of data and drafting the manuscript and figures.

## Conflict of Interest Statement

The authors declare that the research was conducted in the absence of any commercial or financial relationships that could be construed as a potential conflict of interest.
